# The effect of combined maxillary pad movable appliance and FR-III functional appliance in the treatment of skeletal Class III malocclusion of deciduous teeth

**DOI:** 10.1186/s12903-022-02547-x

**Published:** 2022-11-11

**Authors:** Lihua Lyu, Huidong Lin, Hua Huang

**Affiliations:** 1 Department of Stomatology, The Children’s Hospital, Zhejiang University School of Medicine, National Clinical Research Center for Child Health, Hangzhou, Zhejiang China; 2 Department of Pediatric Dentistry, College of Stomatology, Guangxi Medical University, Nanning, Guangxi China

**Keywords:** Deciduous tooth stage, Skeletal Class III, Maxillary pad movable appliance, FR-III functional appliance, Cephalometric measurement

## Abstract

**Background:**

To evaluate the therapeutic effect of maxillary pad movable appliance combined with FR-III functional appliance in treating skeletal Class III malocclusion of deciduous teeth and provide a reference for optimizing clinical treatment methods.

**Methods:**

A total of 30 pediatric patients were randomly selected between April 2012 and April 2019. They were in stage IIA osseous skeletal Class III malocclusion, treated with maxillary pad movable appliance to relieve the reverse, combined with FR-III functional appliance to maintain a median relationship to stage IIIA. A self-control study of children before and after treatment was used, and paired t-test was used to evaluate the changes in the measurement indexes of the IIA and IIIA stage X-rays and changes in the bone and soft tissue profiles.

**Results:**

After 3 years of treatment, SNA, ANB, and NA-PA in the sagittal osteofacial index of the jawbones increased, SNB decreased, and the Y-axis angle in the vertical index of the jawbones increased. U1-SN, U1-NA, U1-NA distance, L1-MP, L1-NB, and L1-NB distance in the index of labial inclination of upper and lower central incisors increased, while U1-L1 decreased. The sagittal anomalies of the jawbones were improved, and there were significant differences before and after treatment (*P* < 0.05). FCA, ULP, and UL-EP increased, soft-tissue facial prominence and facial height increased, and the relationship between the upper lip and the aesthetic plane was harmonious. None of the 30 children with skeletal Class III malocclusion in the deciduous stage experienced recurrence in stage IIIA.

**Conclusions:**

Combined treatment with the maxillary pad movable appliance and the FR-III functional appliance is suitable for children with skeletal Class III malocclusion in the deciduous stage.

## Introduction

Class III malocclusion is one of the common oral malocclusions, it is characterized by hypoplasia of the maxilla, protrusion of the mandible, or dysplasia of the maxilla and mandible, often manifested as anterior crossbite, concave type, Some scholars argue that the risk factors can be divided into two aspects, i.e., genetic and environmental factors or the combination of the two [1–5]. According to the survey results presented by the Orthodontic Professional Committee of the Chinese Stomatological Association in 2000, the prevalence of Class III malocclusion in deciduous teeth is 14.94% [6]. Survey results released by China Health and Nutrition in 2020 revealed that the prevalence rate of Angler type III malformations in deciduous teeth was 13.26% [7].After orthodontic treatment of deciduous teeth, the recurrence rate of anterior crossbite in mixed dentition has been reported to be as high as 46.8% [8]. If the deciduous teeth are not treated, further aggravation during growth and development are likely to happen, mainly resulting in the overburden and abnormal coverage of the anterior teeth and the occurrence of temporomandibular joint disorder syndrome, which can affect the growth and function of the child’s maxillofacial area, and even have adverse effects on mental health [2, 9, 10].

At present, most scholars advocate the early correction of malocclusion [10–13], the early intervention methods for deciduous malocclusion include movable cushion appliance, head cap and chin pocket, maxillary anterior traction, FR-III functional appliance, bracket fixation technology, etc. [14–16], these methods can relieve anterior crossbite, and each has its advantages and disadvantages. It is necessary to select appropriate correction methods according to preoperative diagnosis. The main goal of early treatment of deciduous tooth cross is to relieve the cross and prevent recurrence [9], thus, maintaining and directing the normal growth of the jawbones is the direction of our efforts.

**Fig. 1:**
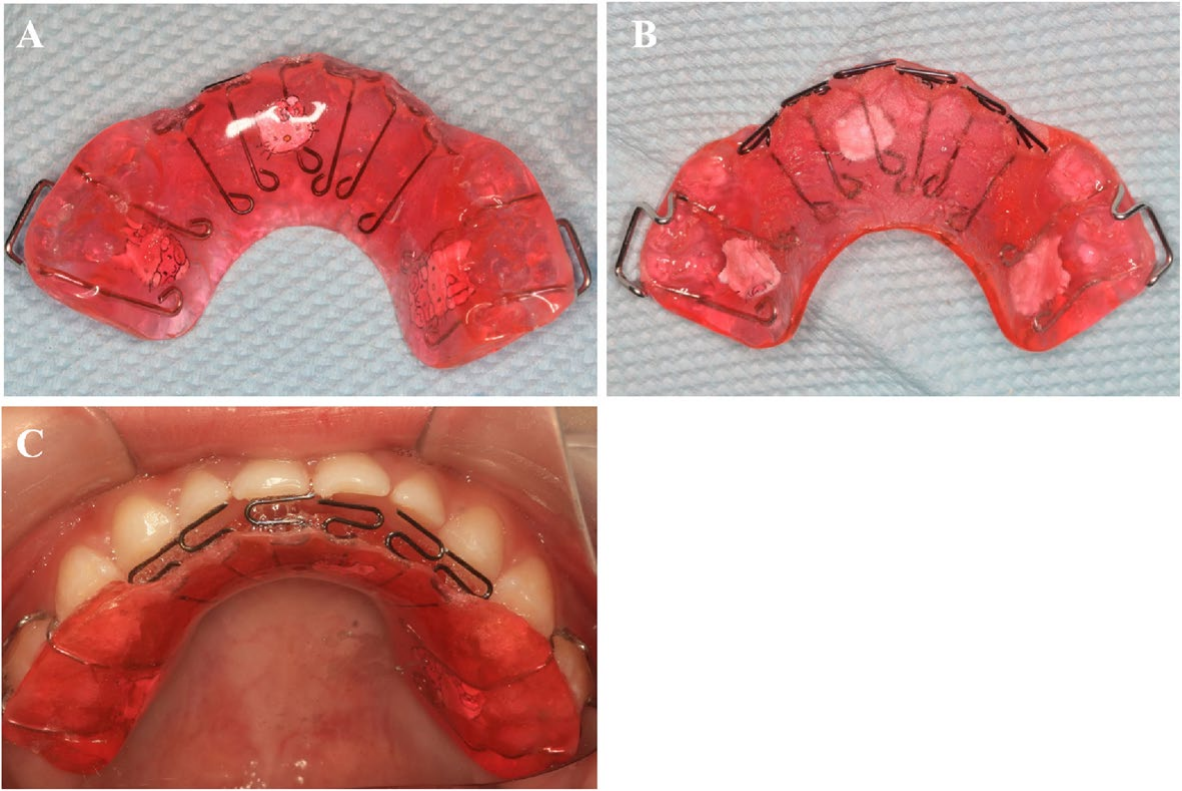
Maxillary pad movable appliance (**A**) Front (**B**) Back (**C**) Intraoral photograph

**Fig. 2:**
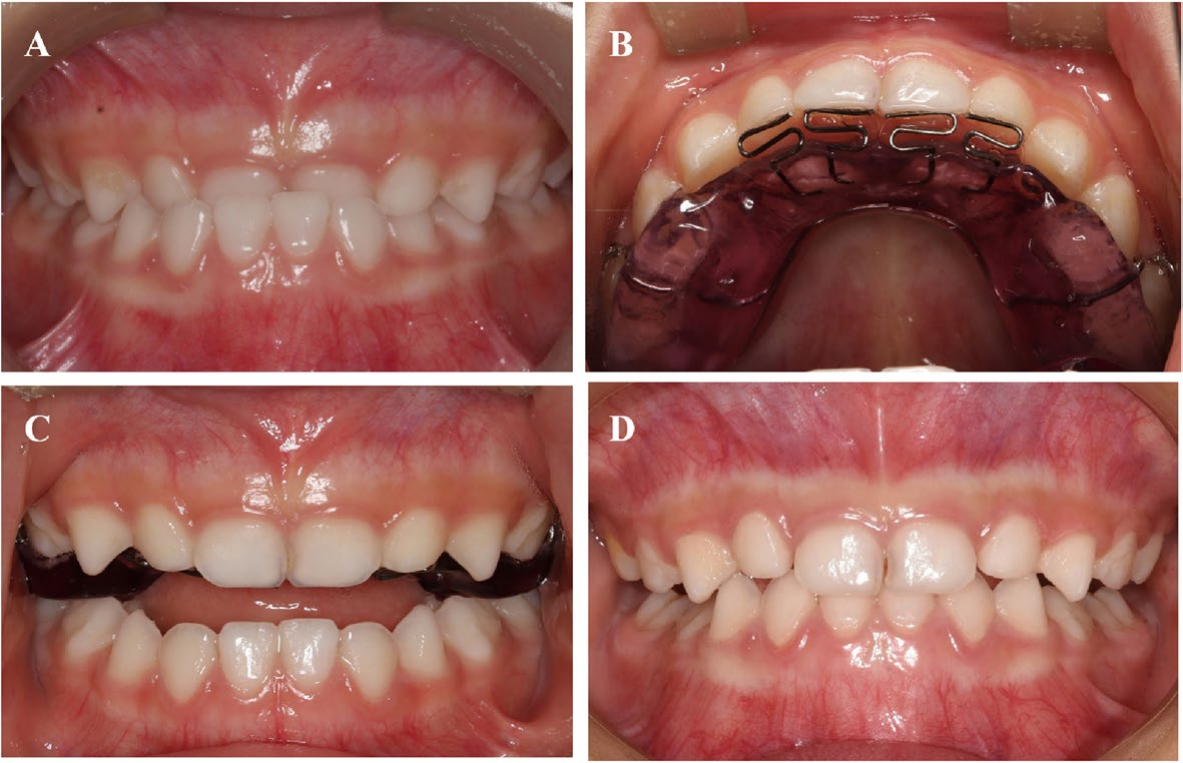
Maxillary pad movable appliance case (**A**) Before treatment (**B**) Initial stage (**C**) Positive bite (**D**) Phased end

**Fig. 3:**
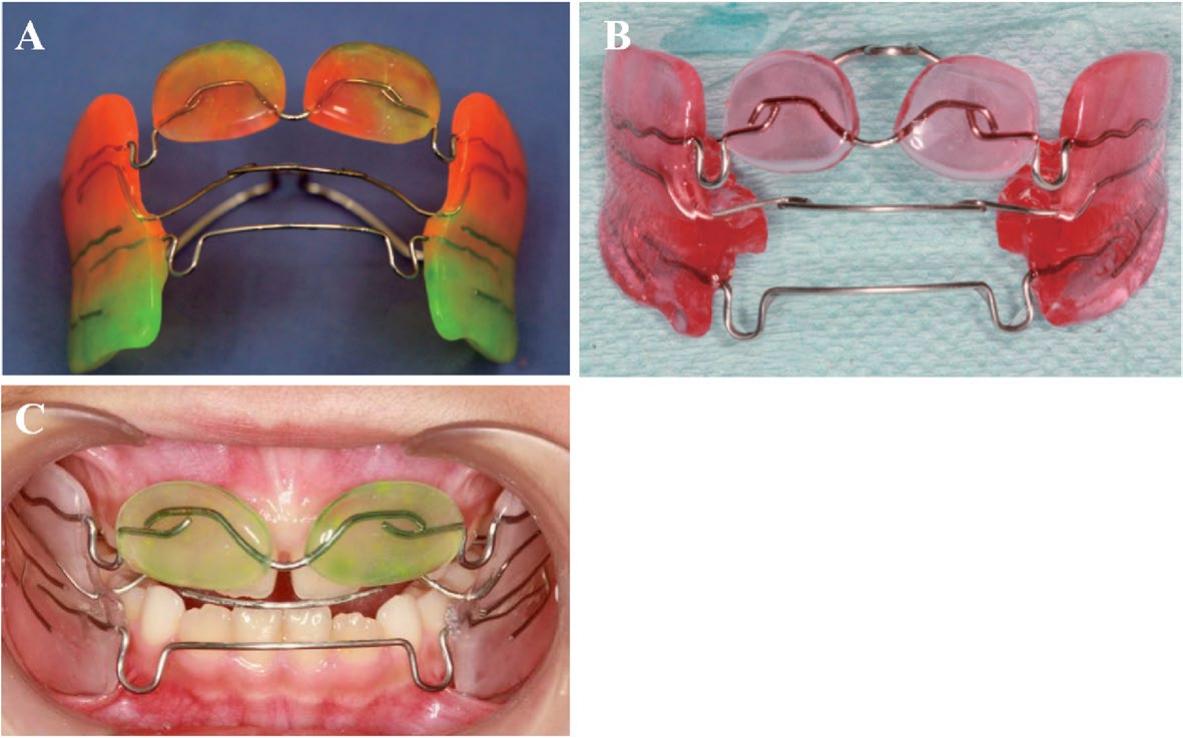
FR-III functional appliance (**A**) Without repositioning occlusal plate (**B**) With repositioning occlusal plate (**C**) Intraoral photograph

In this study, we used the maxillary pad movable appliance (Figs. 1 and 2) to relieve the retroversion and then applied the FR-III functional appliance (Fig. 3) to maintain the centered relationship, combined treatment of skeletal Class III malocclusion in deciduous stage and observation until mixed dentition. Hyperbolic tongue spring of maxillary pad movable appliance increases force on the front teeth with malocclusion, pushing the lateral lip movement of the front teeth with malocclusion, thus changing the lip inclination of the front teeth and the occlusal coverage of the front teeth, using the principle of repositioning occlusal plate, the maxillary pad movable appliance is made to guide the mandibular retreat, as well as relieving malocclusion in a short time. After the malocclusion is removed, the mandibular is moved backward, and the occlusion is reconstructed, the FR-III functional appliance is used to maintain the median relationship and change the muscle function of the oral and maxillary system and the relative position of the mandibular abutments, prevent the recurrence of cross bite during mixed dentition.

The purpose of this study was to investigate the effect of combined maxillary pad movable appliance and FR-III functional appliance in the treatment of skeletal Class III malocclusion of deciduous teeth on the development of craniomaxillofacial hard and soft tissues, guide the normal growth of teeth and jawbones, so as to effectively improve the facial appearance of children with skeletal Class III malocclusion, and provide effective strategies for clinical orthodontic treatment.

## Methods

### Research subjects

Before treatment, all the children and their parents in this study were informed of their condition, treatment plan, treatment time, cost, etc. The children and their parents had informed consent and signed an informed consent form. This retrospective study was approved by the Ethics Committee of Guangxi Medical University (approval number: Shen 20,210,148). Informed consent was obtained from the parents/guardians of the subjects for all studies. Our study have conformed to the STROBE guidelines. We confirm that all methods were carried out in accordance with relevant guidelines and regulations in the declaration. Our study performed in accordance with the Declaration of Helsinki.

The children with end-stage reaching IIIA (complete eruption of first permanent molars/partial or complete eruption of permanent front teeth) stage were recruited from the Department of Pediatric Stomatology, Affiliated Stomatological Hospital of Guangxi Medical University between April 2012 and April 2019. Their initial diagnosis was stage IIA (completion of deciduous teeth occlusion) skeletal Class III malocclusion [4], which was treated with the combination of the maxillary pad movable appliance and the FR-III functional appliance. According to the random number table method, 30 children were randomly selected, including 13 boys and 17 girls. The age at first diagnosis was (5.25 ± 1.25) years old. All the subjects were staged according to the Hellman occlusal development stage [17]; they were recorded as stage IIA before treatment and recorded as stage IIIA after treatment. Inclusion criteria were the following: ① patients first time diagnosed with Class III malocclusion, with 4 or more deciduous anterior teeth crossed; deciduous molars were in a mesial relationship, 4° < ANB < 2.65°, and the mandible could be retracted to the opposite edge; ② the patient was in stage IIA before treatment and in stage 3A at the end of the treatment; ③ no cleft lip and palate deformity; ④ the bilateral temporomandibular joints had good mobility, no snapping and pain, and no obvious asymmetry of facial development; ⑤ no systemic diseases; ⑥ not received orthodontic intervention; ⑦ without protraction, occlusal interference, and tonsil hypertrophy; ⑧ no treatment interruption, all the corrections have been completed to reach stage IIA, and there was a qualified X-ray head lateral radiograph for IIA stage and IIIA stage. Exclusion criteria were: the patient could not cooperate with the treatment due to the young mental age (mental age < 210).

### Treatment method

In stage IIA, the maxillary pad movable orthodontic device, which was worn all day, was utilized to release the reverse, the progress was reviewed monthly. After the anterior teeth overbite and overjet more than 1 mm, adjust the occlusal plate in several times to assist the molar construction. After the release of the reverse, the FR-III functional appliance, which was worn 8 h a day, maintained the neutral relationship until stage IIIA, the progress was reviewed every two months. Take it off when eating, clean the teeth after eating, and then put the appliance in the mouth. Devices were adjusted if uncomfortable or replaced if found unsuitable for wearing. The appliances were all made by the same technician in the laboratory of the Stomatological Hospital Affiliated with Guangxi Medical University. During the correction process, the oral cavity of the children shall be checked at each follow-up visit, and the oral problems shall be handled in a timely manner, such as timely filling of caries, removal of non retained deciduous teeth, retained deciduous teeth, supernumerary teeth, deformed teeth, dental tumors, etc.

### X-ray lateral cephalometric film shooting and measurement of indicators before and after treatment

**Fig. 4:**
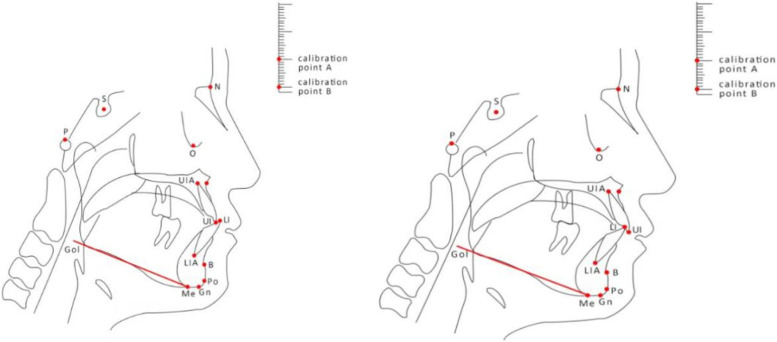
Schematic diagram of the measurement marker points before and after hard tissue treatment. N: nasion point; S: center point of sella; O: orbital point; P: ear point; A: upper alveolar seat point; UI: upper central incisor point; UIA: upper central incisor apex point; B: lower alveolar seat; LI: lower incisor point; LIA: lower incisor apical point; Po: anterior mentation point; Me: submental point; Gn: chin apex, GOI: tangent to the lower edge of the mandibular angle, distance calibration point A, distance calibration point B

**Fig. 5:**
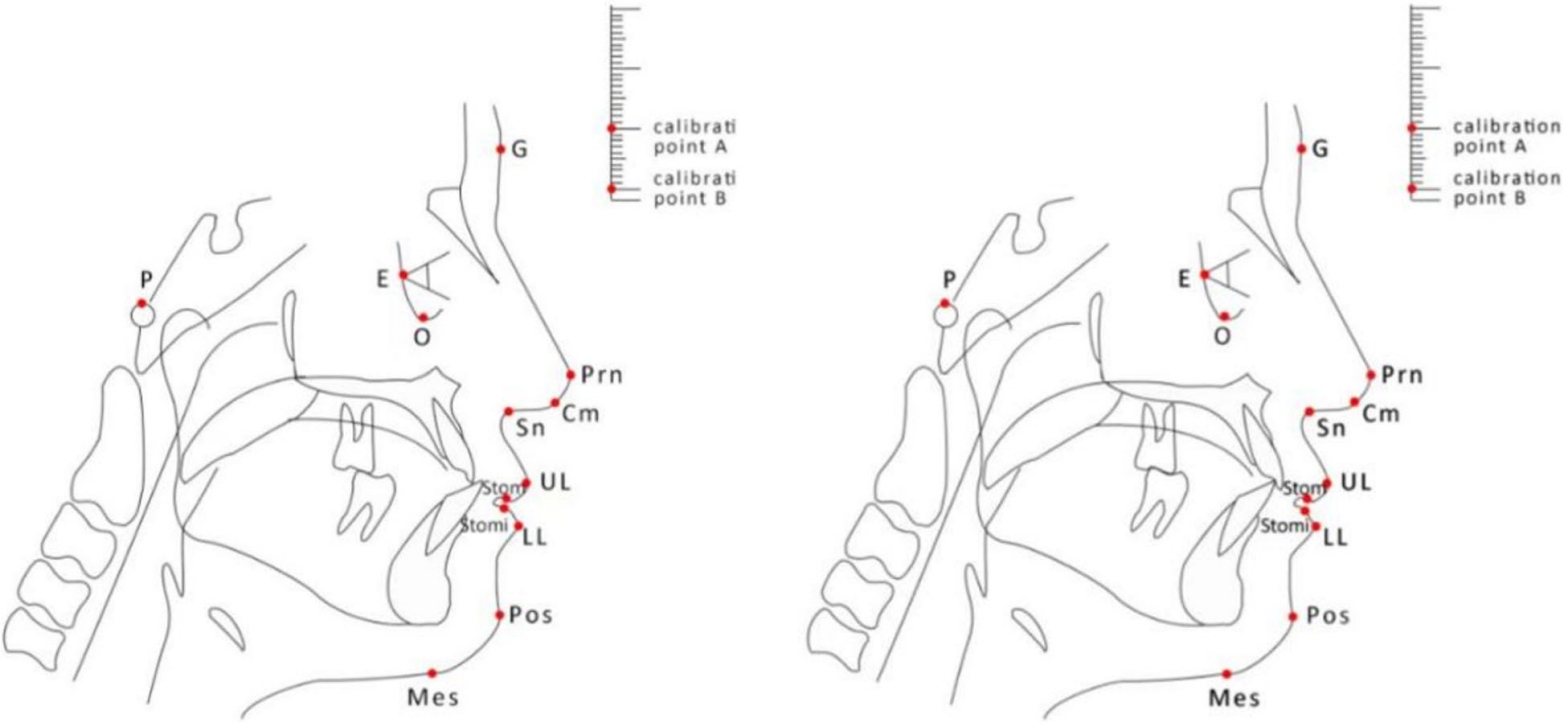
Schematic diagram of measurement landmarks before and after soft tissue treatment. G: Forehead point; E: eye point; Prn: nasal vertex; Cm: columella point; Sn: infranasal point; UL: upper lip protuberance; stoms: upper mouth point; stomi: lower mouth point; LL: lower lip prominent point; Pos: soft tissue premental point; mes: soft tissue submental point; P: ear point; O: orbital point, distance calibration point A, distance calibration point B

All children took panoramic radiograph and X-ray lateral cephalometric film before treatment (stage IIA) and after treatment (stage IIIA). X-ray lateral cephalometric films were taken by the same operator using the same equipment (Orthophos XG Plus DS Ceph, Sirona, Germany). The ground was parallel, the mandible was in a staggered position of the cusps, the facial muscles were relaxed, and the soft and hard tissues were clearly outlined. Indexes were measured at intervals of 6 months, and all measurement indexes were analyzed by cephalometric analysis using Beijing Medical University’s clinical analysis method [2]. There were 27 measurement indexes of craniomaxillofacial hard tissue (Fig. 4, Tables 1, 2, and 3) and soft tissue (Fig. 5 and Table 4). In the cephalometric measurement project, the unit of angle was degree (°), and the unit of line distance was millimeter (mm); each index was measured 3 times, and the average value was taken.

**Table 1:** Sagittal measurement indexes and annotations of the jaw

Metric	Notes
SNA(°)	The angle formed by S–N-A
SNB(°)	The angle formed by S–N-B
ANB(°)	The angle formed by A-N-B
NP-FH(°)	The posterior lower intersection angle of the intersection of the face plane NP (the line connecting N and Po) and the eye-ear plane FH (the line connecting P and O)
NA-PA(°)	The corner of the N to A connection (NA) and the extension of the Po to A connection (PA)

**Table 2:** Measurement indexes and annotations of the vertical orientation of the jaw

Metric	Notes
MP-SN(°)	The intersection angle of the mandibular plane MP (the line tangent to the lower edge of the mandibular angle through Me) and the plane of the anterior skull base (the line connecting S and N)
FH-MP(°)	The intersection angle between the FH plane and the MP plane
Y-axis angle(°)	The lower front corner where the line connecting S and Gn (SGn) intersects with FH
Po-NB(mm)	The vertical distance from Po to N-B line
SN-FH(°)	N point variation comparison value, the angle between the SN plane and the FH plane

**Table 3:** Measurement indexes and annotations of upper and lower anterior teeth

Metric	Notes
U1-SN(°)	The inferior inner angle where the long axis of the upper central incisor (UI-UIA) intersects the SN plane
U1-NA(°)	The angle between the long axis of the upper central incisor (UI-UIA) and the line connecting the NA
U1-NA distance (mm)	The vertical distance from UI to NA
U1-L1(°)	UI and UIA are connected, LI and LIA are connected; the back angle of the intersection of the two lines
L1-MP(°)	Upper medial angle where the long axis of the lower central incisor (LI-LIA) intersects the mandibular plane MP
L1-NB(°)	The angle between the long axis of the lower central incisor (LI-LIA) and the line connecting the NB
L1-NB distance(mm)	Vertical distance from LI to NB connection

**Table 4:** Soft tissue measurement indicators and annotations

Metric	Notes
NLA(°)	The front cross angle of Sn and Cm connection and Sn and UL connection
FCA(°)	The back angle of the line connecting G and Sn and the line connecting Sn and Pos
UFH(%)	Draw a vertical line from E and Sn to the G-Sn line, and the distance between the two vertical lines
ULL(%)	Sn and Stoms are perpendicular to the Sn-Pos line; the distance between the two perpendiculars
LLL(%)	Mes and Stomi draw perpendiculars to the Sn-Pos line; the distance between the two perpendiculars
ULP(mm)	UL to Sn-Pos connection distance
LLP(mm)	LL to Sn-Pos connection distance
UL-EP(mm)	Distance from UL to Prn-Pos
LL-EP(mm)	Distance from LL to Prn-Pos
Z angle(°)	The line connecting the Pos with the prominence of the lips (upper or lower lip) and the angle between the orbital ear plane (FH)

UFH (%) + ULL(%) + LLL(%) = 100%

### Statistical analysis

SPSS 22.0 (IBM, Stanford, USA) statistical software was used for data analysis. The count data were expressed by [*n*(%)], and the test method was χ2. The measurement data were expressed by (*x* ± *s*), and the test method was tested by *t*. *P* < 0.05 indicated statistically significant difference. The self before and after control study and paired t-test were used to evaluate the changes in stage IIA and IIIA measurement indexes of the research subjects and evaluate the bone and face shape and soft tissue of their X-ray lateral cephalometric film changes in profile. The test level α = 0.05, i.e., *P* < 0.05 indicated statistically significant difference.

## Results

**Fig. 6:**
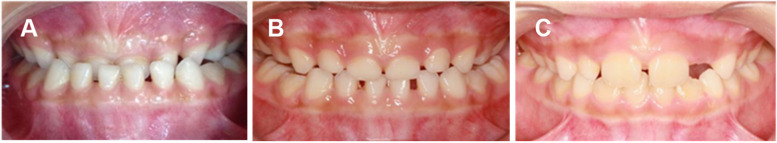
(**A**) Before, (**B**) during, (**C**) late stages of treatment. Before treatment: initial stage, during treatment: removal of anti-occlusion, late treatment: end of treatment

The average treatment time of 30 children with stage IIA to IIIA was 3 years (Fig. 6), and their ages ranged from (5.25 ± 1.25) years to (8.25 ± 1.25) years. By combining axillary pad movable appliance with FR-III functional appliance in the treatment of skeletal Class III malocclusion of deciduous teeth, the comparative analysis of the cephalometric data of stage IIA and IIIA showed that the sagittal anomaly of the jawbone was significantly improved, just as the labial inclination of the upper and lower anterior teeth and the profile of soft tissue. There was no reverse recurrence in any of the 30 children in stage IIIA stage.

### Comparison of sagittal index of jawbones

After treatment, the index of maxillary position SNA increased by 1.29° on average (*P* < 0.05), and the sagittal bone mass of the maxilla increased. SNB, an index of mandibular position, decreased on average by 0.71° (*P* < 0.05), and the mandible was relatively retracted. The sagittal relationship index ANB of the maxilla and maxilla increased by 1.98° on average, and the maxillary relative protrusion index NA-PA increased by 1.93° on average; the observed difference was statistically significant (*P* < 0.05). On the contrary, the facial angle NP-FH, an index of mandibular retraction, decreased by an average of 0.73°; the difference was not statistically significant (*P* > 0.05) (Table 5).

**Table 5:** Changes in sagittal indexes before and after treatment in 30 children with skeletal Class III malocclusion in deciduous teeth

Indicator	IIA stage	IIIA stage	IIA-IIIA stage	*t*	*P*
SNA(°)	79.53 ± 5.17	80.82 ± 4.15	-1.29 ± 2.03	-3.479	0.002**
SNB(°)	79.71 ± 3.65	78.99 ± 3.70	0.72 ± 1.83	2.151	0.040*
ANB(°)	-0.16 ± 1.47	1.83 ± 1.39	-1.98 ± 1.61	-6.735	0.000***
NP-FH(°)	83.96 ± 3.03	83.23 ± 4.22	0.73 ± 3.19	1.249	0.222
NA-PA(°)	2.02 ± 4.04	3.95 ± 3.76	-1.93 ± 4.06	-2.602	0.014*

^*^p < 0.05; **p < 0.01; ***p < 0.001

### Comparison of vertical index of jawbones

Compared with stage IIA and IIIA, the Y-axis angle of the chin development index increased by 1.86° on average (*P* < 0.05), and the chin was well developed. Po-NB distance increased by 0.71 mm on average, FH-MP increased by 0.76° on average, MP-SN decreased by 0.31° on average, and SN-FH increased by 0.86° on average, with no statistical significance (*P* > 0.05) and no vertical change (Table 6).

**Table 6:** Changes in vertical indexes before and after treatment in 30 children with skeletal Class III malocclusion in deciduous teeth

Indicator	IIA stage	IIIA stage	IIA-IIIA stage	*t*	*P*
MP-SN(°)	36.46 ± 5.72	36.15 ± 5.87	0.31 ± 3.21	0.523	0.665
FH-MP(°)	30.88 ± 4.87	31.64 ± 4.13	-0.76 ± 2.35	-1.777	0.086
Y-ais angle(°)	63.02 ± 2.71	64.88 ± 3.06	-1.86 ± 2.43	-4.199	0.000***
Po-NB distance(mm)	-1.02 ± 1.91	-0.31 ± 1.01	-0.70 ± 2.03	-1.893	0.068
SN-FH(°)	5.42 ± 2.48	4.55 ± 3.44	0.86 ± 2.82	1.674	0.105

^*^p < 0.05; **p < 0.01; ***p < 0.001

### Comparison of labial inclination index of upper and lower anterior teeth

Compared with before treatment, the labial inclination index U1-SN of upper anterior teeth increased by 17.24° on average (*P* < 0.05), and the upper anterior teeth had labial inclination after orthodontic treatment. U1-NA increased on average 15.35° (*P* < 0.05), the inclination and prominence of the upper incisors increased; the U1-NA distance was close to the normal value in stage IIIA, with an average increase of 2.75 mm (*P* < 0.05), and the prominence of the upper incisors was close to normal, indicating upper incisor teeth had labial inclination after treatment. Compared with stage IIA, the labial inclination index L1-MP of lower anterior teeth increased by 8.07° on average (*P* < 0.05), and the lower anterior teeth were still lingually inclined after treatment but improved compared with before treatment. L1-NB increased by 7.24° on average (*P* < 0.05). *P* < 0.05). The inclination and prominence of the lower anterior teeth were still insufficient after orthodontic treatment, but they were improved compared with those before the operation. The L1-NB distance increased by an average of 2.05 mm in stage IIIA (*P* < 0.05). The inclination and prominence of lower anterior teeth were improved compared with the preoperative state. The upper and lower anterior dental arch indexes U1-L1 were significantly improved in stage IIIA, with an average decrease of 25.53° (*P* < 0.05), indicating that the upper and lower incisor protrusions were more coordinated after orthodontic treatment (Table 7).

**Table 7:** Changes in labial inclination index of upper and lower anterior teeth before and after treatment in 30 children with skeletal Class III malocclusion in the deciduous stage

Indicator	IIA stage	IIIA stage	IIA-IIIA stage	*t*	*P*
U1-SN(°)	91.87 ± 6.12	109.12 ± 6.01	-17.24 ± 7.51	-12.584	0.000***
U1-NA(°)	12.58 ± 7.01	27.93 ± 5.65	-15.35 ± 8.32	-10.106	0.000***
U1-NA distance(mm)	1.71 ± 2.03	4.48 ± 1.35	-2.75 ± 2.40	-6.270	0.000***
U1-L1(°)	152.77 ± 5.19	127.25 ± 8.22	25.53 ± 11.25	12.426	0.000***
L1-MP(°)	79.19 ± 5.19	87.26 ± 7.10	-8.07 ± 6.13	-7.215	0.000***
L1-NB(°)	15.21 ± 5.28	22.45 ± 6.13	-7.24 ± 5.88	-6.741	0.000***
L1-NB distance(mm)	2.03 ± 1.18	4.08 ± 1.83	-2.05 ± 1.15	-9.774	0.000***

^*^p < 0.05; **p < 0.01; ***p < 0.001

### Comparison of soft tissue profile indicators

Compared to before, after the correction, the soft tissue profile index FCA changed from (4.00 ± 3.44)° to (8.05 ± 4.14)°, revealing an average increase of 4.01° within the normal range (*P* < 0.05). The concave type was improved. The upper lip protrusion index was (4.87 ± 1.50) mm in stage IIA and (6.51 ± 2.01) mm in stage IIIA, with an average increase of 1.64 mm (*P* < 0.05). The protrusion of the upper lip was improved but still insufficient. The upper lip and aesthetic plane index UL-EP changed from (-0.10 ± 1.88) mm to (1.64 ± 2.05) mm, with an average increase of 1.74 mm (*P* < 0.05). The distance from the upper lip protrusion to the E line improved, while NLA, UFH, ULL, LLL, LLP, LL-EP, and Z angle changes revealed no statistical significance (*P* > 0.05) (Table 8).

**Table 8:** Changes of soft tissue profile indexes before and after treatment in 30 children with skeletal Class III malocclusion of deciduous teeth

Indicator	IIA stage	IIIA stage	IIA-IIIA stage	*t*	*P*
NLA(°)	100.58 ± 11.33	99.31 ± 11.31	1.27 ± 11.31	0.615	0.543
FCA(°)	4.00 ± 3.44	8.05 ± 4.14	-4.06 ± 3.56	-6.240	0.000***
UFH(%)	41.43 ± 3.08	41.30 ± 2.70	-0.13 ± 2.53	0.281	0.781
ULL(%)	19.24 ± 1.65	19.61 ± 1.67	-0.37 ± 2.27	-0.893	0.379
LLL(%)	39.33 ± 3.08	40.06 ± 4.33	-0.73 ± 4.41	-0.902	0.374
ULP(mm)	4.87 ± 1.50	6.51 ± 2.01	-1.64 ± 1.86	-4.818	0.000***
LLP(mm)	5.21 ± 1.77	5.55 ± 1.90	-0.34 ± 2.06	-0.914	0.368
UL-EP(mm)	-0.10 ± 1.88	1.64 ± 2.05	-1.74 ± 1.56	-6.127	0.000***
LL-EP(mm)	2.51 ± 1.91	2.77 ± 2.20	-0.26 ± 2.43	-0.578	0.568
Z angle(°)	69.71 ± 7.88	66.74 ± 12.96	2.98 ± 12.96	1.168	0.252

^*^p < 0.05; **p < 0.01; ***p < 0.001

## Discussion

The comprehensive skeletal, dentaland esthetics cephalometric evaluation is the key point to the success of the result [18]. During the 3-year treatment period, the statistical analysis of X-ray lateral cephalometric film data before and after the treatment of the children, the study showed that SNA (+ 1.29°), ANB (+ 1.98°), and NA-PA(+ 1.93°) in sagittal bone surface indexes of the upper and lower jawbones increased, while SNB (-0.71°) decreased, the sagittal anomalies of the jawbones were improved, and there were significant differences before and after treatment (*P* < 0.05). The present study showed that the improvement of the sagittal skeletal shape of the maxilla and mandible might be derived from the force, mechanical force, muscle strength, growth, and development, as well as the combined action of the maxillary pad movable appliance and the FR-III functional appliance. The FR-III functional appliance did not recur with the growth and development after the guided treatment, thus suggesting that the sagittal skeletal profile of the children with deciduous tooth osseous inversion was improved after orthodontic treatment. In his study, Miyajima K [19] conducted a long-term follow-up study on 1376 untreated patients with Class III malocclusion, finding that the improvement of sagittal skeletal shape in children was due to the correction rather than their own growth and development, which is consistent with the conclusions of the present study. Yang et al. [20] showed that the FR-III functional appliance in the treatment of Class III malocclusion patients during the growth and development period did not stimulate the maxilla bone development in the short-term (2–3 years) and long-term (8–9 years), but inhibited mandibular development. Lip block of FR-III functional appliance can relieve the inhibition of the upper lip on the development of the upper jawbone, and as the buccal screen is in direct contact with the lower teeth/alveolar, it can continue to inhibit the development of the lower jawbone and maintain the effect achieved at the previous stage of anti-orthodontic treatment [21].

The Y-axis Angle of the vertical jaw index increased (+ 1.86°), the chin was well developedand no vertical change. Data analysis in this study proved that the FR-III functional appliance had a relative improvement effect on patients within three years, meaning that the maintenance effect of the FR-III functional appliance was effective.

Alexander et al. [22] argued that the angle of the mandibular plane angle does not change significantly during the growth and development of untreated patients with Class III malocclusion. U1-SN (+ 17.24°), U1-NA (+ 15.35°), U1-NA distance (+ 2.75 mm), L1-MP (+ 8.07°), L1-NB (+ 7.24°), and L1-NB distance (+ 2.05 mm) in the lip inclination index of the upper and lower middle incisor. U1-L1 decreased (-25.53°), and the difference was statistically significant before and after treatment (*P* < 0.05). This study used the FR-III functional appliance without repositioning occlusal plate for the low-angle and equal-angle cases. FR-III functional appliance with repositioning occlusal plate was used for high-angle cases, i.e., the principle of positioning the occlusal plate was applied, and padding was added to achieve vertical control. Therefore, the improvement of the labiolingual inclination of the upper and lower anterior teeth observed in the study subjects may result from the combined effect of the maxillary pad movable appliance and the FR-III functional appliance. None of the 30 children with Class III malocclusion of deciduous teeth had a recurrence in stage IIIA, indicating that maxillary pad movable appliance combined with FR-III functional appliance can effectively improve the facial shape of children with Class III malocclusion, guide the normal growth of jawbone, and provide an effective strategy for clinical orthodontic treatment.

The increase of FCA, ULP, and UL-EP, and the increase in soft tissue surface protrusion and surface height, confirmed that the relationship between the upper lip and the aesthetic plane was harmonious. After orthodontic treatment, the protruding degree of soft tissue was improved, and the degree of the upper lip was improved, but the change in the degree of the lower lip was not obvious. The improvement of facial soft tissue was mainly due to the change in the degree of protrusion of the upper lip, whose length did not significantly change. This result is consistent with a number of previous studies [23, 24]. Japanese scholars performed a long-term follow-up of patients with reversal during the growth and developmental period, finding that the upper lip receded from IIA to IIIA, and the protrusion of the lower lip did not significantly change [19]. Based on these research results, it can be inferred that the corrective effect can improve the soft tissue side. The changing trend in face shape due to soft tissue is consistent with the change in ANB, while the change in lower lip protrusion was not obvious, and there was inconsistency with the changes in bone-associated face shape. Soft tissue could compensate to a certain extent for the bone shape, and the results of this experiment are consistent with the related research on face shape, bone shape, and soft tissue shape [25–28].

In the present study, we innovative used the maxillary pad movable appliance to design repositioning occlusal plate and the traditional maxillary reed device so as to improve the sagittal bone and occlusal relationship in children and at the same time to release the locking relationship with the opposing teeth, as well as mandibular guide retraction by positioning the occlusal plate for occlusal reconstruction. The sagittal anomalies of the jawbon and occlusal relationship of children were improved, and effectively adjust the overbite and overjet. The FRII functional appliance is used to maintain the median relationship to the IIIA stage, provide a good environment for the normal development of the jawbones, guide the normal growth of the jawbones, and improve the facial shape. This approach alleviates the concerns of children and their families to a certain extent and increases confidence in the therapeutic effect [29].There was no recurrence during the study period, indicating that this method can provide a new method and idea for the correction of skeletal class III malocclusion. However, for children with skeletal Class III malocclusion, it should be observed until the early adulthood. The object of this study is currently in Phase IIIA, which is the clinical effect observation of staged treatment of skeletal malocclusion in deciduous teeth, and the conclusions drawn are staged conclusions.

## Conclusions

Combined maxillary pad movable appliance and FR-III functional appliance in the treatment of skeletal class III malocclusion of deciduous teeth, the comparison of the lateral cephalometric films before and after the treatment showed that the maxilla continued to develop, the mandibular development was limited, and the sagittal index of maxilla was improved, the chin continued to develop, and there was no significant change in vertical index of jawbone, the labial inclination of upper and lower anterior teeth and soft tissue profile were improved. All children with skeletal crossbite in the deciduous period had no recurrence from the treatment to the first permanent molar construction stage and the permanent anterior teeth eruption stage. The jawbones were in a normal growth and development state. This study obtained a more positive conclusion.

Stage IIA to VA (the completion of the third permanent molars eruption) are all periods of correction, maintenance and observation. The correction from IIA to IIIA is only staged. The children with skeletal factors still need further correction after IIIA. We hope that through continuous optimization, we can expect to get a better correction strategy for skeletal Class III malocclusion. The maintenance after the removal of malocclusion is also an important part of the treatment of skeletal Class III malocclusion. In the future research, the author will review regularly according to the different stages of the children’s treatment, record and deal with the problems in the review process in a timely manner, to further explore the methods of maintaining and guiding the development of the jawbones after the removal of skeletal class III malocclusion.

## Data Availability

All data generated or analyzed during this study are included in this published article.
